# Base of tongue volume and dose as predictors of late dysphagia after definitive or adjuvant radiotherapy for oral cavity squamous cell carcinoma

**DOI:** 10.1016/j.phro.2026.101038

**Published:** 2026-07-12

**Authors:** Joseph Azria, Arnaud Beddok, Esteban Brenet, Delphine Antoni, Laurence Auzeau, Nathaniel Assouly, Marine Fontaine, Sofiane Guendouzen, Jean-Claude Merol, Yacine Merrouche, Philippe Guilbert, Antonio Da Silva Ribeiro Mota

**Affiliations:** a Department of Radiation Oncology, Institut Godinot, Reims, France; b Head and Neck Surgery Department, Centre Hospitalo-Universitaire Reims, France; c Radiotherapy Department, Paul Strauss Cancer Center, Strasbourg, France; d Department of Oncology, Institut Godinot, Reims, France; e ENT department, Jean-Godinot Institut, Cancer Centre, Reims, France

**Keywords:** Radiotherapy, Base of Tongue, Dysphagia, Radiation-induced side effects, Artificial Intelligence

## Abstract

**Background and purpose:**

Radiation-induced dysphagia is a major determinant of quality of life in patients treated for oral cavity squamous cell carcinoma (OCSCC). While dose to the pharyngeal constrictor muscles has been extensively investigated, the base of tongue (BOT) remains poorly studied. This study aimed to evaluate the association between BOT volume and dose–volume parameters and late dysphagia.

**Material and methods:**

Fifty-two patients with OCSCC treated with volumetric modulated arc therapy (VMAT) between 2019 and 2023 were retrospectively analyzed. All patients had a prophylactic feeding tube (FT) placed before VMAT. The base of tongue was delineated using an artificial intelligence-based segmentation algorithm and reviewed by a senior radiation oncologist. Late dysphagia was defined as prolonged FT dependency using the cohort-specific 75th percentile (> 442.5 days). Volumetric and dose/volume parameters of the BOT (D_0%–_D_100%_) were analyzed using logistic regression, receiver operating characteristic (ROC) curve analysis, and Kaplan–Meier methods.

**Results:**

The median BOT volume for the entire cohort was 18.4 cm^3^ (interquartile range [IQR]: 15.7–21.3 cm^3^). Patients with late dysphagia had smaller BOT volumes than those without (15.9 vs. 19.5 cm^3^, *p* = 0.02). A BOT volume < 19.12 cm^3^ was associated with increased dysphagia risk. No dose/volume parameter reached statistical significance, although a trend suggested lower risk for a BOT D_50%_ < 52.2 Gy (*p* = 0.21). A combined model integrating BOT volume and D_50%_ achieved an area under the curve (AUC) of 0.74 (95% CI: 0.61–0.87).

**Conclusions:**

Smaller BOT volume was associated with increased risk of prolonged FT dependency after radiotherapy for OCSCC. BOT dose metrics were not significant but showed hypothesis-generating trends that warrant validation in larger cohorts and dose-optimization studies.

## Highlights

• Smaller BOT volume was associated with prolonged FT dependency.
• BOT volume < 19.1 cm^3^ identified patients at higher dysphagia risk.
• AI-based segmentation enabled standardized BOT delineation.
• Combined BOT volume and D50 achieved an AUC of 0.74.
• BOT metrics may support future swallowing-sparing RT strategies.

## Graphical abstract

**Figure:**
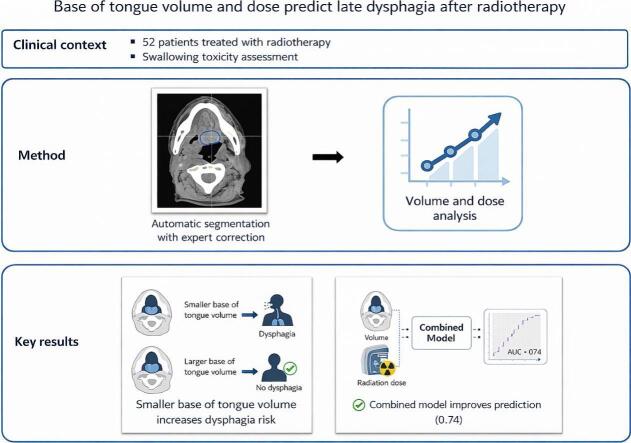

## 1 Introduction

Oral cavity squamous cell carcinoma (OCSCC) accounted for an estimated 389,846 new cases and 188,438 deaths globally in 2022, according to the World Health Organization (WHO) [1]. Radiation therapy (RT), often combined with surgery and/or chemotherapy, is a standard treatment modality for OCSCC [2]. Despite its efficacy in achieving local tumor control and improving survival, RT is associated with adverse effects, including late dysphagia, which significantly affects nutrition and quality of life [3]. Late dysphagia, reported in 33–40% of patients following RT for OCSCC, is defined as swallowing dysfunction occurring beyond 6 months after treatment and is attributed to post-radiation soft tissue fibrosis that impairs muscle contractility, resulting in persistent swallowing dysfunction [4], [5], [6].

Previous studies have established associations between RT dose to swallowing-related structures, such as the supraglottic region, larynx, and pharyngeal constrictor muscles, and the risk of post-treatment dysphagia in OCSCC patients [7], [8], [9]. For instance, dysphagia-optimized intensity-modulated radiotherapy (DO-IMRT) has been shown to reduce the incidence of swallowing dysfunction by limiting the dose to these structures [10].

The base of the tongue (BOT) is a key anatomical component in the swallowing process, playing a central role in bolus manipulation and propulsion during deglutition. Its coordination with the palate and pharyngeal muscles ensures effective swallowing and prevents aspiration. The genioglossus and geniohyoid muscles contribute to bolus propulsion, while the BOT's structural integrity is critical for maintaining swallowing function [11]. Consequently, the extent of BOT involvement is carefully considered in surgical planning to preserve swallowing outcomes.

In the context of RT, the BOT is vulnerable to radiation-induced damage, including inflammation, edema, and fibrosis, which compromise tongue strength and coordination, contributing to dysphagia [12], [13], [14].

While the relationship between RT dose to other swallowing structures and dysphagia is well-documented, limited data are available regarding the impact of BOT dose and volume on late dysphagia, defined in our study as prolonged FT dependency. In addition, accurate delineation of the BOT remains challenging in routine practice, and variability in contouring may affect both volumetric and dose/volume analyses, highlighting the importance of standardized imaging-based segmentation in studies investigating dose–effect relationships. This study aimed to address this gap by investigating the relationship between BOT volumetric and dose/volume parameters and late dysphagia, as assessed by feeding tube (FT) dependency duration.

## 2 Materials and methods

### 2.1 Patients and treatment characteristics

This study was conducted as an observational, retrospective, single-center analysis. Fifty-two patients aged over 18 years with histologically confirmed OCSCC treated at Institut Godinot between May 2019 and August 2023 were included. Eligible participants underwent RT or chemoradiotherapy targeting the primary tumor or as an adjuvant to surgery. The presence of an FT before the start of treatment was also an inclusion criterion. During the study period, prophylactic FT placement before RT was part of the institutional standard of care for OCSCC patients. The removal of an FT was decided during routine follow-up visits according to the treating physician's clinical judgment and institutional practice. FT dependency duration was calculated from the documented dates of FT placement and removal. As the removal of an FT could be influenced by follow-up schedules and clinical practice patterns, the recorded duration should be interpreted as an estimate of the actual time to swallowing recovery. Patients treated with palliative intent, those with a history of previous RT, chemotherapy, or head and neck cancers, were excluded. All patients underwent a planning computed tomography (CT) scan in the treatment position to ensure accurate RT targeting. Treatment planning strictly adhered to established dose constraints and institutional protocols [15]. The RT was delivered using volumetric modulated arc therapy (VMAT) according to institutional planning protocols. Patients were included through a non-opposition process after receiving an information letter. The study was conducted in accordance with the Declaration of Helsinki and French regulations governing retrospective observational research (MR-004 framework). All patients were informed of the use of their data for research purposes and were given the opportunity to object to participation.

### 2.2 Data recording

Comprehensive clinical data were collected for all patients, including World Health Organization (WHO) performance status, body weight measurements before RT and during follow-up (1, 3, 6, 12, and 24 months), gender, tumor–node–metastasis (TNM) classifications, including clinical and pathological (cTNM and pTNM), FT placement and removal dates, RT start and end dates, and delivered RT dose. Toxicities were recorded according to the Common Terminology Criteria for Adverse Events (CTCAE) version 5 classification [16], encompassing acute toxicities such as dysgeusia, dysphagia, xerostomia, and mucositis, as well as late toxicities such as fibrosis. These data were gathered during routine follow-up visits scheduled every three months during the first two years post-treatment and every six months thereafter for up to five years.

### 2.3 Delineation and dose/volume data collection

**Fig. 1:**
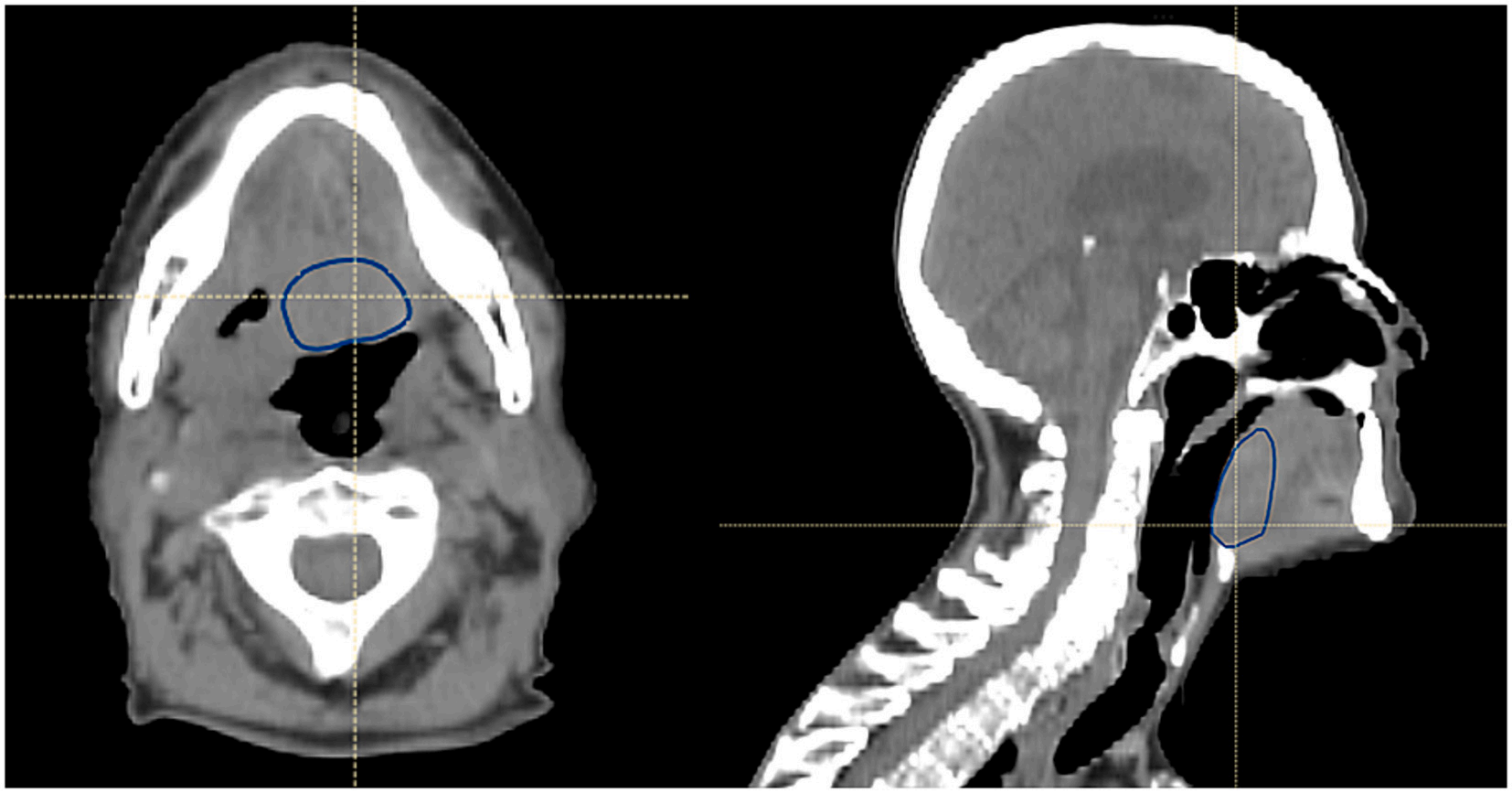
Delineation of the base of tongue on planning CT images. Axial (left) and sagittal (right) views are shown, with the base of tongue contoured in blue. Contours were initially generated using a deep learning–based auto-segmentation tool and subsequently reviewed and manually corrected slice-by-slice by a senior radiation oncologist according to published anatomical guidelines, before volumetric and dose/volume analyses. (For interpretation of the references to colour in this figure legend, the reader is referred to the web version of this article.)

The BOT delineation was performed on the original planning CT scans using RayStation software (RaySearch Laboratories AB, Stockholm, Sweden) (Fig. 1). Initial contours were generated using the RayStation deep learning autosegmentation module and were subsequently reviewed and manually corrected slice-by-slice by a senior radiation oncologist (ASM). Particular attention was paid to maintaining midline symmetry and excluding adjacent structures such as the glossotonsillar sulcus, vallecula, and pharyngeal mucosa when not corresponding to intrinsic tongue musculature. Anatomical boundaries were defined according to published swallowing organ-at-risk recommendations [17], including cranial and caudal limits at C1 and the hyoid bone, respectively, with exclusion of adjacent non-intrinsic tongue structures. When available, magnetic resonance images (MRI) were reviewed to support anatomical interpretation and ensure consistency of BOT delineation. Other organs at risk, including the pharyngeal constrictor muscles and parotid glands, had already been delineated during routine treatment planning, in accordance with institutional practice and published guidelines. Dose/volume parameters extracted for the BOT included volume, mean dose, maximum dose (D_1%_), minimum dose (D_99%_), and dose–volume metrics (D_0%_–D_100%_). Mean doses to the larynx, pharyngeal constrictor muscles, and parotid glands were also recorded, along with standard planning target volume (PTV) dose/volume parameters (D_95%_, D_max_, D_2%_).

### 2.4 Statistical analysis

The primary endpoint for distinguishing patients with and without late dysphagia was the duration of FT dependency, defined as the time interval between FT placement and removal. Late dysphagia was operationally defined as prolonged FT dependency, using the cohort-specific 75th percentile (> 442.5 days) to identify the longest-duration group. Patients were categorized based on the 75th percentile of FT duration: those at or below this threshold were classified as having no late dysphagia, while those above it were classified as having late dysphagia. This threshold was chosen because no validated cut-off for prolonged FT dependency exists in the literature, and the upper quartile approach is commonly used in exploratory clinical studies to identify patients with the most severe or prolonged functional impairment while preserving statistical power. A sample size calculation was conducted to evaluate the relationship between BOT radiation dose and dysphagia severity. Based on an assumed 19% increase in dysphagia risk per 10 Gy increment [7], and applying a significance level of 0.05 with a power of 0.9, a sample size of 26 subjects per group was estimated. Descriptive analyses summarized categorical variables as percentages and continuous variables as medians with interquartile ranges (IQRs), with 95% confidence intervals. Non-parametric tests were used for group comparisons, including the Wilcoxon Mann-Whitney test for continuous variables and Fisher's exact test for categorical variables. Statistical analyses were conducted using RStudio (version 4.1.2). The pROC package was used to calculate the area under the curve (AUC) with corresponding 95% confidence intervals. Association analyses to identify predictors of severe dysphagia, including BOT dose/volume parameters, were performed using chi-square tests and logistic regression models. Additional analyses were performed to assess the potential impact of prior surgery. Stratified analyses were conducted according to treatment modality (radiotherapy alone versus surgery followed by radiotherapy), and interaction terms between BOT volume and surgical status were tested in logistic regression models.

## 3 Results

The demographic and clinical characteristics are summarized in Table 1. The median age was 65.3 years (IQR: 57.8–73.7). At diagnosis, the median weight was 66.6 kg (IQR: 57.75–75 kg), and the median weight loss during treatment was 2 kg (IQR: 0–5 kg). Smoking status was distributed as follows: 9 never smokers, 22 former smokers, and 21 current smokers. The tumor locations were the mobile tongue (17 patients), the floor of the mouth (16 patients), the pelvic-lingual region (7 patients), the gingiva-mandibular region (4 patients), the retromolar trigone (3 patients), the gum (3 patients), and the inner cheek (2 patients). The median RT dose was 66 Gy (IQR: 66–69.6 Gy). Surgery was performed before RT in 39 patients (75%), with the most common procedure being pelvi-mandibulectomy (12 patients). Concomitant chemotherapy with cisplatin was administered to 36 patients.

**Table 1:** Patient and treatment characteristics.

	Number	Percentage
**Sex**		
Male	33	63
Female	19	37
**Age (median, IQR)**	65.3 years (IQR: 57.8–73.7)	

**WHO**		
0	17	33
1	28	54
2	7	13
3	0	0

**Surgery**		
Yes	39	75
No	13	25
**cT**		
1	2	4
2	7	13
3	17	33
4	26	50
**cN**		
0	22	42
1	9	18
2	20	38
3	1	2
4	0	0

**Concurrent chemotherapy**		
Yes	36	69
No	16	31

**Primary site**		
Mobile tongue	17	33
Floor of the mouth	16	31
Pelvic-lingual region	7	13
Gingiva-mandibular region	4	8
Retromolar trigone	3	6
Gum	3	6
Inner cheek	2	3
**Dose (Median, IQR)**	66 Gy (IQR: 66–69.6 Gy)	

The median follow-up time was 14.5 months (IQR: 8.25–23). The median duration of FT dependency was 273.5 days (IQR: 176–442.5 days). Thirteen patients (25%) experienced late dysphagia, defined as FT dependency exceeding 442.5 days. Acute toxicities included dysphagia in 52% of patients (27/52) at grade 2 and in 35% (18/52) at grade 3, mucositis in 46% (24/52) at grade 2 and 27% (14/52) at grade 3, and dermatitis in 41% (21/52) at grade 2 and 3% (2/52) at grade 3. Xerostomia occurred in 12% of patients (6/52) at grade 2 and in 6% (3/52) at grade 3. No grade 4 acute toxicities were reported. Late toxicities included xerostomia in 10% of patients (5/52) at grade 2 and 8% (4/52) at grade 3, and dysphagia in 2% (1/52) of patients at both grade 2 and grade 3. Dysgeusia occurred in 2% of patients (1/52) at grade 2, with no grade 3 cases. Fibrosis was not reported at grade 2 or grade 3. No grade 4 late toxicities were documented. A summary of acute and late toxicities is presented in Table 2.

**Table 2:** Acute and late toxicities.

Toxicities	Grade 0	Grade 1	Grade 2	Grade 3
**Acute**				
Dysphagia	3 (6%)	4 (7%)	27 (52%)	18 (35%)
Dysgeusia	52 (100%)	0 (0%)	0 (0%)	0 (0%)
Mucositis	3 (6%)	11 (21%)	24 (46%)	14 (27%)
Dermatitis	3 (6%)	26 (50%)	21 (41%)	2 (3%)
Xerostomia	35 (68%)	8 (14%)	6 (12%)	3 (6%)

**Late**				
Fibrosis	50 (97%)	2 (3%)	0 (0%)	0 (0%)
Xerostomia	32 (63%)	11 (21%)	5 (10%)	4 (8%)
Dysphagia	48 (93%)	2 (3%)	1 (2%)	1 (2%)
Dysgeusia	51 (98%)	0 (0%)	1 (2%)	0 (0%)

The median BOT volume for the entire cohort was 18.4 cm^3^ (IQR: 15.7–21.3 cm^3^). Patients with late dysphagia had significantly smaller BOT volumes than those without late dysphagia (16.1 cm^3^ [IQR: 14.9–18.5] vs 19.5 cm^3^ [IQR: 15.8–22.3], Mann–Whitney *U* test, *p* = 0.02). Larger BOT volumes (> 19.12 cm^3^) were associated with a reduced risk of late dysphagia (p = 0.02). Among the cohort, 21 patients had a BOT volume exceeding 19.12 cm^3^ (Fig. S1). Other non-dosimetric variables did not show significant associations with late dysphagia (Mann–Whitney U or Fisher's exact tests as appropriate). These included age (*p* = 0.8), smoking status (NS), concomitant chemotherapy (NS), tumor location (anterior versus posterior/lateral sites) (*p* = 0.31), WHO performance status (*p* = 0.49), and tumor stage (cT) (*p* = 0.65). Tumor stage was also not associated with BOT volume. Median BOT volume was 19.3 cm^3^ (IQR: 16.7–22.2) in cT4 tumors and 16.8 cm^3^ (IQR: 14.9–19.6) in cT1–3 tumors (Mann–Whitney *U* test, *p* = 0.098). Prior surgery was associated with smaller BOT volumes (median 17.8 cm^3^ vs 20.3 cm^3^, Mann–Whitney U test, *p* = 0.038). Consistent with this finding, 26 of 31 patients (84%) with BOT volumes ≤19.12 cm^3^ had undergone surgery, compared with 13 of 21 patients (62%) with BOT volumes >19.12 cm^3^. However, surgery itself was not associated with late dysphagia (Fisher's exact test, *p* = 1.00). Stratified analyses according to treatment modality (radiotherapy alone versus surgery followed by radiotherapy) showed similar patterns between BOT volume and late dysphagia in both subgroups, although statistical significance was not reached within each subgroup. No significant interaction was observed between BOT volume and surgical status (*p* = 0.22), suggesting that the association between BOT volume and late dysphagia was independent of prior surgery.

**Fig. 2:**
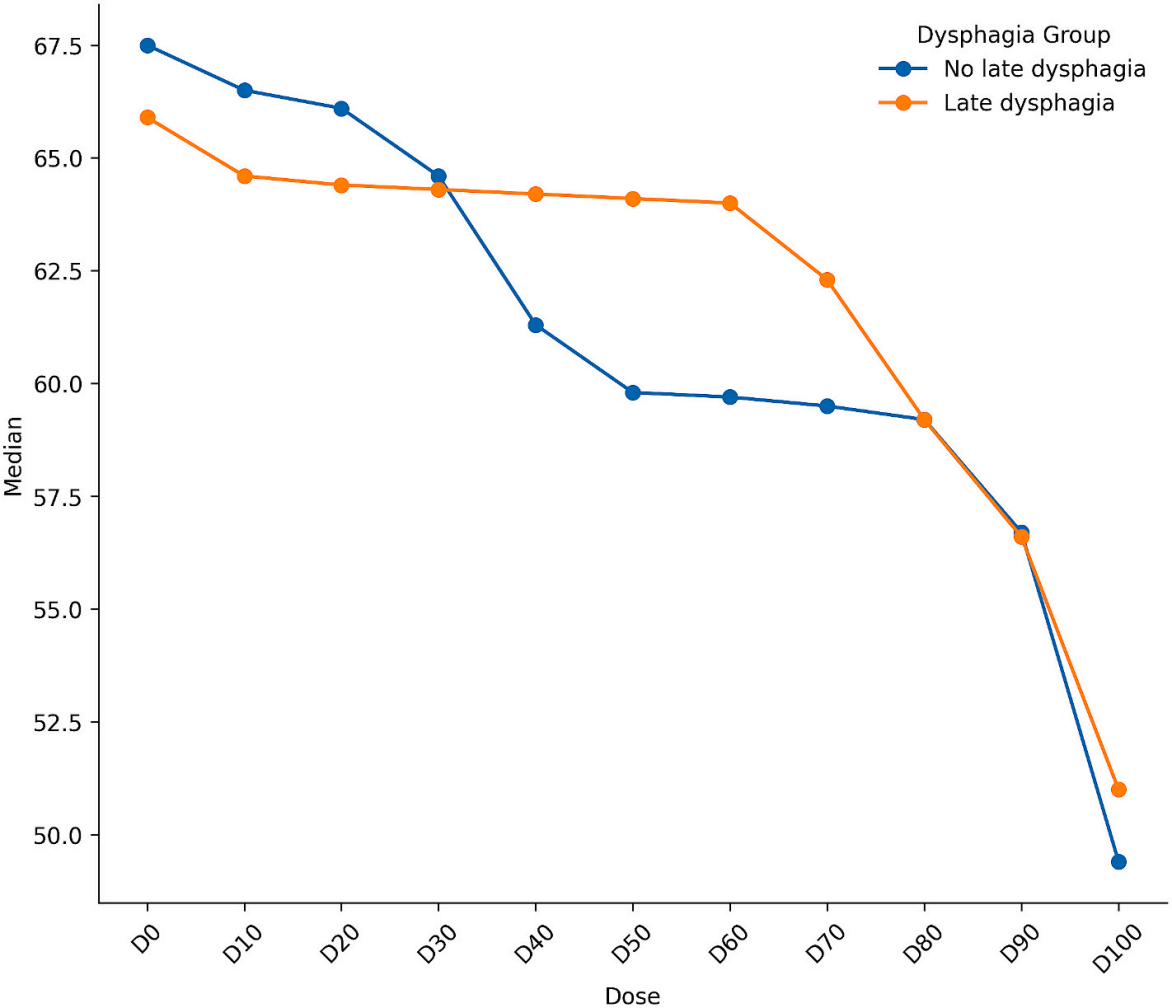
Dose–volume parameters of the base of tongue in patients with and without late dysphagia. Curves represent median dose values (D0%–D100%) for each group.

**Fig. 3:**
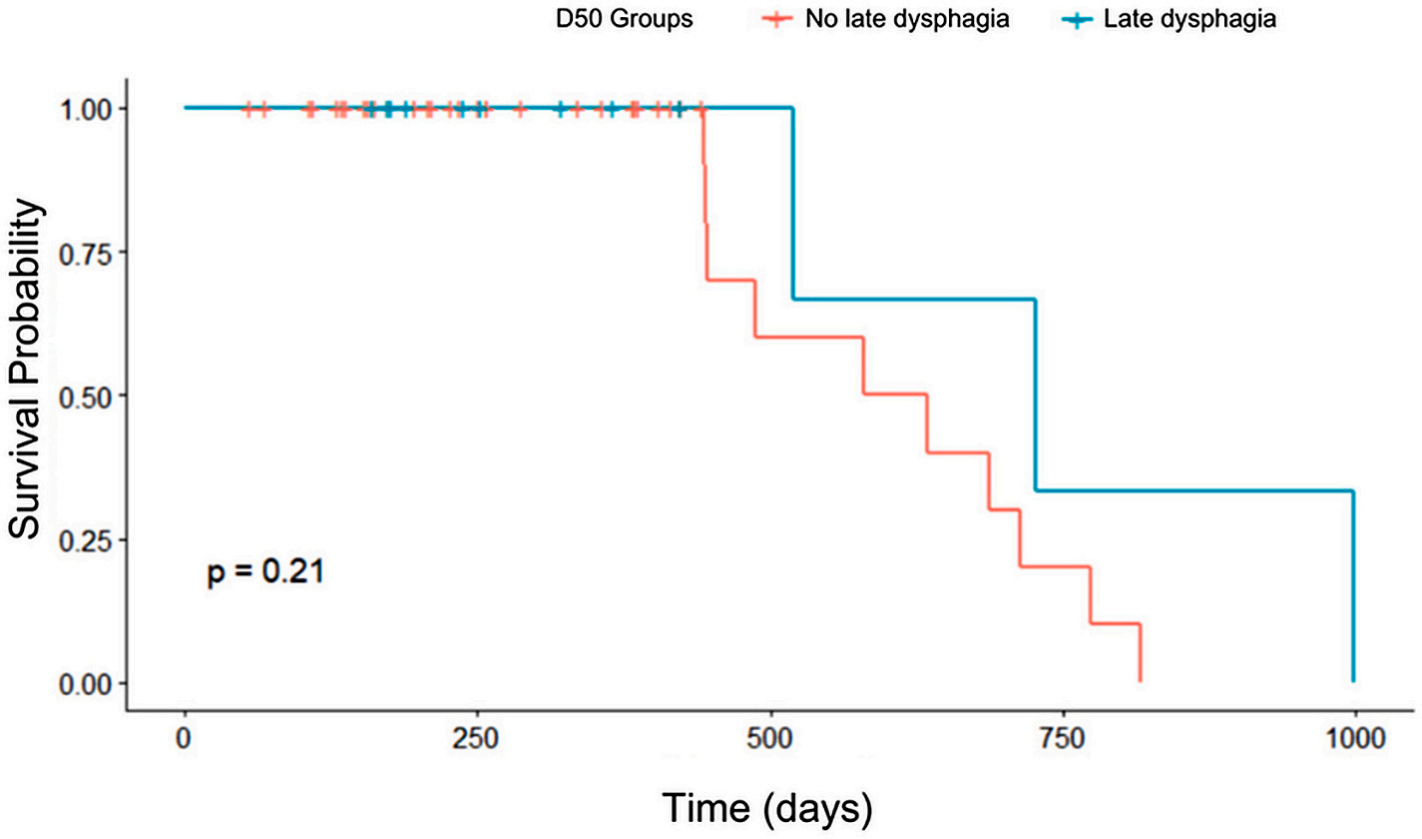
Kaplan–Meier curves showing the probability of remaining free from late dysphagia according to base of tongue D_50%_ dose groups. Tick marks indicate censored observations, and the *p*-value was calculated using the log-rank test.

Univariate analyses did not identify statistically significant associations between BOT dose/volume parameters and late dysphagia (Table S1). The median D_50%_ to the base of tongue was 60.3 Gy (IQR: 52.7–65.7) for the whole cohort. The median D_50%_ was 59.9 Gy (IQR: 51.8–66.1) in patients without late dysphagia and 64.0 Gy (IQR: 52.9–65.5) in patients with late dysphagia, with no significant difference between groups (Mann–Whitney *U* test, *p* = 0.97) (Fig. 2). Kaplan–Meier analysis identified a threshold of 52.2 Gy for the D_50%_ to the BOT as a potential reference point for distinguishing patients at higher risk of late dysphagia. Patients receiving doses above this threshold showed a higher estimated risk of late dysphagia, although the difference was not statistically significant (log-rank test, *p* = 0.21) (Fig. 3).

**Fig. 4:**
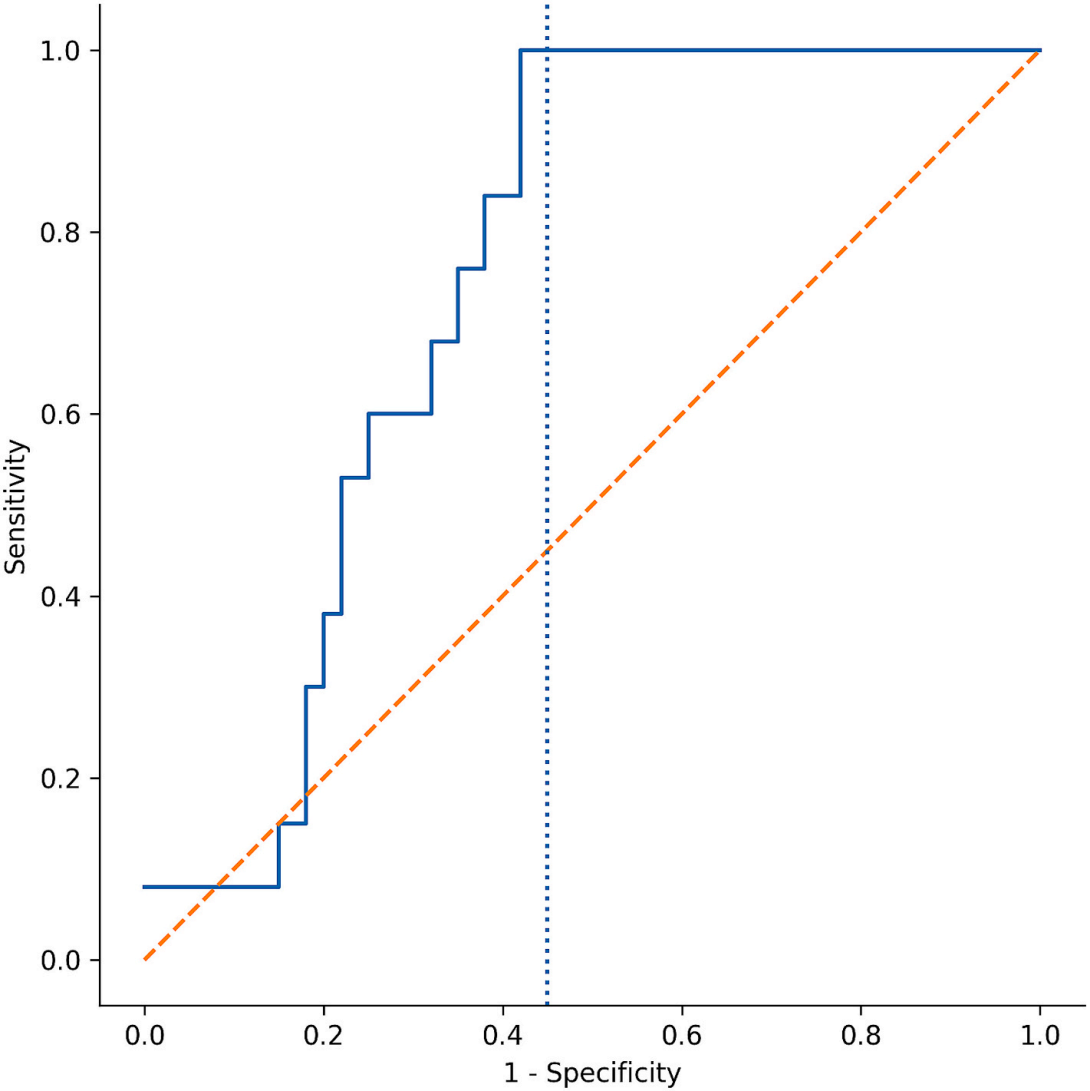
Receiver operating characteristic (ROC) curve of the multivariate logistic regression model predicting late dysphagia based on base of tongue volume and D_50%_. The dashed line indicates the selected D_50%_ threshold.

A multivariable logistic regression model incorporating BOT volume and D_50%_ was developed to predict late dysphagia. The model demonstrated moderate discriminative performance, with an AUC of 0.74 (95% CI: 0.61–0.87) (Fig. 4). A significant association was observed between larger BOT volumes (> 19.12 cm^3^) and a reduced risk of late dysphagia (*p* = 0.02), suggesting a protective role for increased BOT volume. Although higher D_50%_ values showed a trend toward an increased risk of late dysphagia, the association was not statistically significant (*p* = 0.37).

## 4 Discussion

This study explored the relationship between the volumetric and dose/volume parameters of the BOT and the occurrence of late dysphagia in patients with OCSCC treated using VMAT. The analysis revealed that smaller BOT volumes were significantly associated with a higher risk of late dysphagia. While no dose/volume parameter reached statistical significance, a non-significant trend was observed with higher D_50%_ doses to the BOT and increased dysphagia risk. To further assess the interplay between these factors, a multivariable logistic regression model combining BOT volume and D_50%_ was developed, demonstrating moderate predictive accuracy for late dysphagia (AUC: 0.74).

Late dysphagia is a common and significant complication in head and neck oncology, with important impacts on patient nutrition, quality of life, and recovery. Numerous studies have highlighted the relationship between radiation dose to swallowing-related structures and the risk of late dysphagia [8], [18], [19], [20], [21] (Table S2). A phase 3 multicenter randomized trial compared dysphagia-optimized IMRT (DO-IMRT) to standard IMRT, showing that limiting the mean dose to the pharyngeal constrictor muscles to below 50 Gy reduced swallowing dysfunction without compromising oncological efficacy [10]. Similarly, a prospective study demonstrated a dose–response relationship, showing that doses exceeding 60 Gy to the pharyngeal constrictor muscles and larynx were associated with severe dysphagia and aspiration. These associations were evaluated using videofluoroscopy and clinical assessments [13]. Our findings align with these studies but provide a novel perspective by focusing on the BOT, a structure essential for swallowing mechanics but often overlooked in dose optimization strategies. In this study, a smaller BOT volume (< 19.12 cm^3^) was significantly associated with an increased risk of late dysphagia (*p* = 0.02), emphasizing the importance of volumetric parameters. Although no dose/volume parameter reached statistical significance, a threshold of 52.2 Gy for the D_50%_ dose to the BOT was identified, with a trend toward increased dysphagia risk in patients receiving higher doses. These results support the inclusion of both volumetric and dose/volume factors in risk assessments and treatment planning.

The base of tongue plays a central role in swallowing, contributing to bolus manipulation, propulsion, and coordination with other swallowing structures. In our cohort, patients with smaller BOT volumes were at higher risk of late dysphagia, consistent with surgical practices that prioritize BOT preservation to maintain function. Ear, nose, and throat surgical approaches often assess the percentage of BOT involvement to optimize oncological resection while minimizing functional impairment [22]. The observed protective role of larger BOT volumes aligns with the concept of functional swallowing units, which extends the focus beyond traditional organs at risk to include muscles involved in specific functions, such as hyolaryngeal elevation and tongue base retraction. Reducing radiation doses to the thyrohyoid (< 40 Gy) and genioglossus muscles (< 30 Gy mean dose) has been associated with improved swallowing outcomes [21]. These findings emphasize the need to consider both anatomical and functional aspects of the BOT and related structures in treatment planning.

In the present study, artificial intelligence (AI) was used for BOT delineation. Deep learning–based auto-segmentation was systematically reviewed and corrected when necessary by a senior radiation oncologist, allowing standardization of the delineation process across patients. This is particularly relevant for structures such as the BOT, where anatomical boundaries are less clearly defined and inter-observer variability in manual contouring may affect both volumetric measurements and dose–volume parameters, thereby impacting dose/volume analyses and study reproducibility [23]. Beyond improving efficiency, the use of AI-based segmentation may contribute to improving the consistency of contouring in clinical studies, especially when volumetric endpoints are investigated. For small or irregular swallowing structures such as the BOT, even limited contouring variability may translate into substantial differences in reported dose–volume parameters, further highlighting the importance of standardized delineation methods in dose/volume studies. More broadly, AI applications in radiotherapy are rapidly expanding, particularly in imaging, segmentation, and treatment planning workflows. In this context, AI-enabled standardization may facilitate a more robust integration of imaging, volumetric, and dose/volume data, which is essential for the development of predictive models and individualized treatment strategies [24].

Moreover, although FT dependency is an objective and commonly used endpoint in retrospective studies, its interpretation may be influenced by institutional FT policies and removal practices. As summarized in Table S2, substantial heterogeneity exists across published studies regarding both FT placement strategies and definitions of FT dependency. Some studies relied on predominantly prophylactic FT placement and fixed time-point endpoints such as 12-month FT dependence [18], whereas others reported FT dependency at last follow-up [25], duration of FT use [20], or individualized nutritional recovery criteria [8], [19]. Furthermore, swallowing outcomes have been assessed using a wide range of methodologies, including videofluoroscopy, patient-reported outcome measures, and quality-of-life questionnaires, as summarized in Table S3 [7], [10], [13], [21], [26], [27]. Consequently, direct comparisons of FT dependency rates and durations across studies should be interpreted with caution. In addition, the removal of FT may also be influenced by follow-up schedules and clinical practice patterns, potentially introducing variability. More direct assessments of swallowing function, including videofluoroscopy, manometry, or validated patient-reported outcome measures such as the MD Anderson Dysphagia Inventory (MDADI), could provide complementary information. Therefore, the threshold identified in the present study should be interpreted within the context of a prophylactic FT strategy and may not be directly comparable to thresholds reported in cohorts using different FT management policies.

This study has several limitations that warrant consideration. Although the sample size calculation targeted 90% statistical power, the relatively small cohort of 52 patients may have limited the ability to detect more subtle associations and reduced the generalizability of the findings. Another limitation is that the analysis focused on the base of tongue as a single structure, whereas dysphagia is a multifactorial side effect involving several swallowing-related organs at risk. In addition to treatment-related factors, FT dependency may also be influenced by patient-related and psychosocial factors, including the ability to manage a FT, social support, psychological acceptance, and adherence to swallowing rehabilitation programs. Combining dose–volume parameters from multiple structures may improve predictive performance and should be explored in future work. Finally, dose–volume parameters were derived from planned dose distributions, and anatomical changes during treatment may lead to discrepancies with delivered doses; accumulated dose estimation using deformable image registration could refine this assessment. Despite these limitations, this study provides novel insights into the role of the BOT in radiation-induced dysphagia and supports the integration of volumetric and dose/volume parameters into predictive modeling.

## Data sharing statement

Research data are stored in an institutional repository and will be shared upon request to the corresponding author.

## CRediT authorship contribution statement

**Joseph Azria:** Writing – original draft, Visualization, Investigation, Data curation. **Arnaud Beddok:** Writing – original draft, Visualization, Supervision, Investigation, Formal analysis, Conceptualization. **Esteban Brenet:** Writing – review & editing, Investigation. **Delphine Antoni:** Writing – review & editing, Investigation. **Laurence Auzeau:** Writing – review & editing, Investigation. **Nathaniel Assouly:** Writing – review & editing, Investigation. **Marine Fontaine:** Writing – review & editing, Investigation. **Sofiane Guendouzen:** Writing – review & editing, Investigation. **Jean-Claude Merol:** Writing – review & editing, Investigation. **Yacine Merrouche:** Writing – review & editing, Investigation. **Philippe Guilbert:** Writing – review & editing, Investigation. **Antonio Da Silva Ribeiro Mota:** Writing – review & editing, Validation, Supervision, Methodology, Conceptualization.

## Declaration of competing interest

The authors declare that they have no known competing financial interests or personal relationships that could have appeared to influence the work reported in this paper.

## Appendix A Supplementary data

Supplementary material: This appendix indlude Supplementary Figure S1: Figure S1. Comparison of the base of tongue volume between patients with and without late dysphagia, and three sup. tables.
