# Supplementary material for: Base of tongue volume and dose as predictors of late dysphagia after definitive or adjuvant radiotherapy for oral cavity squamous cell carcinoma
Source: Phys Imaging Radiat Oncol. 2026 Jul 12;40:101038. doi: 10.1016/j.phro.2026.101038 (PMC13449413; doi:10.1016/j.phro.2026.101038)
Supplement: Supplementary file 1 [file mmc1.pdf]

Figure S1. Comparison of the base of tongue volume between patients with and without late dysphagia.

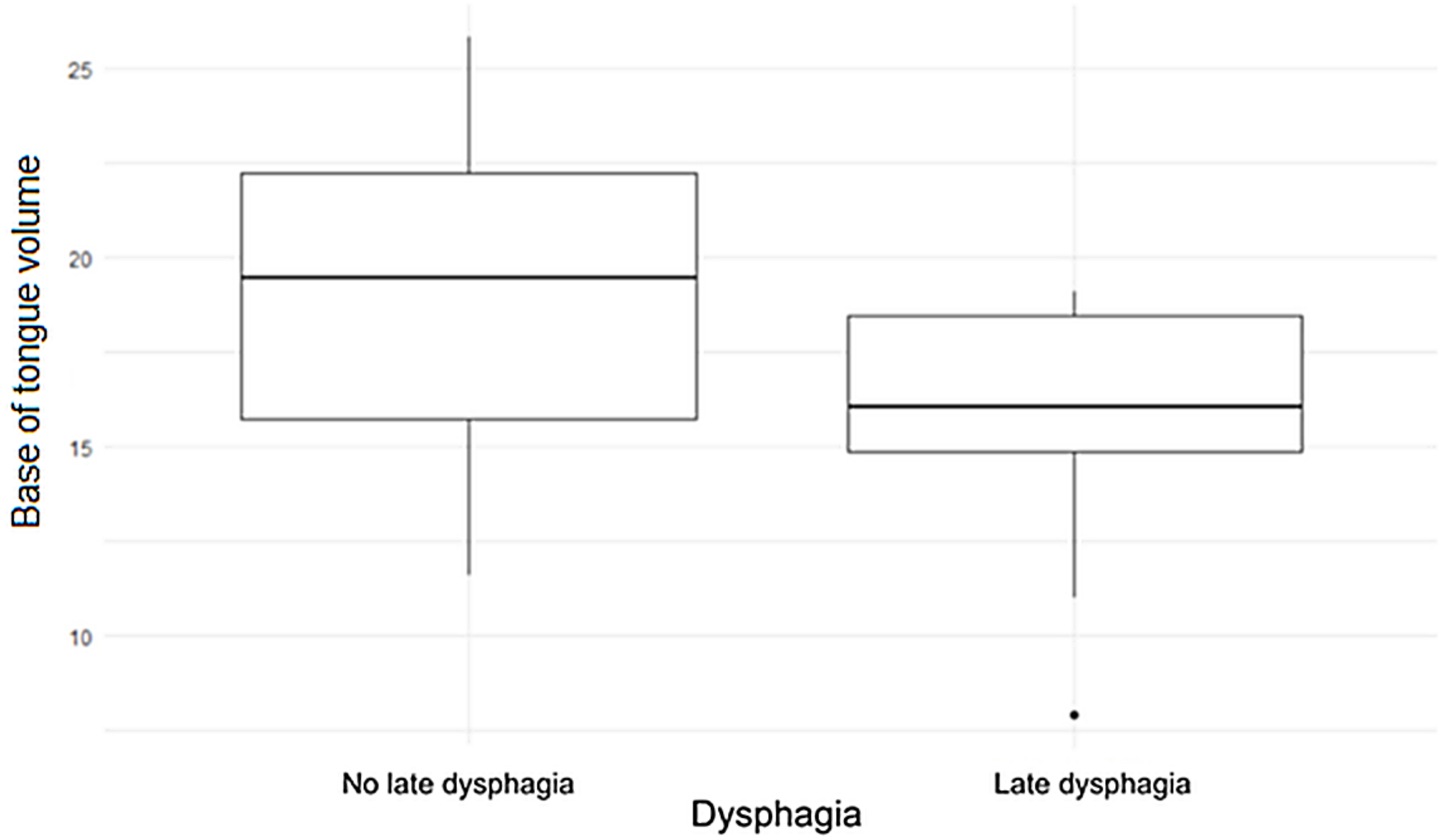

Late dysphagia was defined as prolonged feeding tube dependency occurring beyond 442.5 days after radiotherapy. Boxplots display the median and interquartile range; whiskers indicate the range, and dots represent outliers.

Table S1: Univariate analyses: correlations between the doses received by the BOT and late dysphagia

	Chi Square	P value
BOT Dmin	0,01	0,9
BOT Dmoy	0,06	0,8
BOT Dmax	2,2	0,1
BOT D0cc	2,5	0,1
BOT D10cc	1,4	0,2
BOT D20cc	0,7	0,4
BOT D30cc	0,2	0,6
BOT D40cc	0,1	0,8
BOT D50cc	0,002	0,9
BOT D60cc	0,005	0,9
BOT D70cc	0,01	0,9
BOT D80cc	0,002	1
BOT D90cc	0,1	0,8
BOT D100cc	0,1	0,8

Table S2. Feeding tube management strategies and definitions of gastrostomy dependence in studies evaluating swallowing toxicity

**Study**	**Feeding tube strategy**	**Definition of endpoint**
Ang et al., 2005	Mixed prophylactic and reactive FT placement	FT dependence reported among disease-free survivors at last follow-up
Li et al., 2009	Structured nutritional support with FT	FT discontinued when nutritional and swallowing recovery criteria were met
Caudell et al., 2010	Predominantly prophylactic FT placement before RT	Severe late dysphagia defined as FT dependence at 12 months, aspiration, or pharyngoesophageal stricture requiring dilation
Sanguinetti et al., 2013	Prophylactic FT placement for patients receiving chemotherapy; selective placement for others	FT removed after ≥1 week without use
Vlacich et al., 2014	Reactive FT placement for ≥10% weight loss or when clinically indicated	FT duration defined from insertion to removal; FT removed after ≥3 weeks of adequate oral intake

Abbreviations: FT: feeding tube; RT: radiation therapy.

Table S3. Alternative dysphagia assessment methods used in head and neck radiation therapy studies

**Study**	**Assessment method**	**Endpoint**
Eisbruch et al., 2004	Videofluoroscopy	Aspiration and swallowing dysfunction
Levendag et al., 2007	Videofluoroscopy	Functional swallowing assessment
Kumar et al., 2014	Videofluoroscopy	Objective swallowing dysfunction
Mortensen et al., 2015	EORTC QLQ	Swallowing-related quality of life
Goepfert et al., 2017	MDADI	Patient-reported swallowing outcomes
Nutting et al., 2023	MDADI	Patient-reported swallowing dysfunction

Abbreviations: MDADI, MD Anderson Dysphagia Inventory; EORTC QLQ, European Organisation for Research and Treatment of Cancer Quality of Life Questionnaire; QLQ, Quality of Life Questionnaire.
